# Iron supplementation regulates the progression of high fat diet induced obesity and hepatic steatosis via mitochondrial signaling pathways

**DOI:** 10.1038/s41598-021-89673-8

**Published:** 2021-05-24

**Authors:** Naho Kitamura, Yoko Yokoyama, Hiroki Taoka, Utana Nagano, Shotaro Hosoda, Tanon Taworntawat, Anna Nakamura, Yoko Ogawa, Kazuo Tsubota, Mitsuhiro Watanabe

**Affiliations:** 1 Systems Biology Program, Graduate School of Media and Governance, Keio University, 5322, Endo, Fujisawa, Kanagawa 252-0882 Japan; 2 Health Science Laboratory, Keio Research Institute at SFC, 5322, Endo, Fujisawa, Kanagawa 252-0882 Japan; 3 Department of Environment and Information Studies, Keio University, 5322, Endo, Fujisawa, Kanagawa 252-0882 Japan; 4 Department of Ophthalmology, Keio University School of Medicine, 35 Shinanomachi, Shinjuku-ku, Tokyo, 160-8582 Japan; 5 Department of Internal Medicine, Keio University School of Medicine, 35 Shinanomachi, Shinjuku-ku, Tokyo, 160-8582 Japan

**Keywords:** Iron, Nutrient signalling, Obesity, Endocrine system and metabolic diseases

## Abstract

Disruption of iron metabolism is closely related to metabolic diseases. Iron deficiency is frequently associated with obesity and hepatic steatosis. However, the effects of iron supplementation on obesity and energy metabolism remain unclear. Here we show that a high-fat diet supplemented with iron reduces body weight gain and hepatic lipid accumulation in mice. Iron supplementation was found to reduce mitochondrial morphological abnormalities and upregulate gene transcription involved in mitochondrial function and beta oxidation in the liver and skeletal muscle. In both these tissues, iron supplementation increased the expression of genes involved in heme or iron–sulfur (Fe–S) cluster synthesis. Heme and Fe–S cluster, which are iron prosthetic groups contained in electron transport chain complex subunits, are essential for mitochondrial respiration. The findings of this study demonstrated that iron regulates mitochondrial signaling pathways—gene transcription of mitochondrial component molecules synthesis and their energy metabolism. Overall, the study elucidates the molecular basis underlying the relationship between iron supplementation and obesity and hepatic steatosis progression, and the role of iron as a signaling molecule.

## Introduction

Iron homeostasis, a complex process that manifests as iron accumulation or deficiency, is often associated with metabolic diseases when disrupted. Iron deficiency, an aspect of iron metabolism disruption, is a global epidemic^1^, which poses a concern for metabolic abnormalities that affect approximately 25% of the world population. Similarly, obesity and associated hepatic steatosis are a global issue which increases the risk of developing chronic diseases such as non-alcoholic steatohepatitis (NASH), cardiovascular disease, type-2 diabetes mellitus (T2DM), retinopathy, and certain cancers^2^. Iron deficiency is positively associated with obesity and hepatic steatosis^3,4^. The reduction in obesity improves iron status^5,6^. Furthermore, iron deficiency causes an increase in hepatic lipogenesis and deteriorates systemic lipid homeostasis^7,8^. Evidently, co-occurrence of iron deficiency, obesity, and hepatic steatosis is more than mere coincidence; rather, they are molecularly related and influence each other^9^.

Several studies suggest that mitochondrial dysfunction might be one of the underlying central factors that is associated with iron deficiency^10,11^, obesity^12^, and hepatic steatosis^13^. Iron deficiency decreases gene expressions associated with beta-oxidation in mitochondrial-rich liver and skeletal muscle^8^, which are important target tissues in the prevention and treatment of obesity and hepatic steatosis. However, the mechanism through which iron regulates mitochondrial-related energy metabolism in obesity and hepatic steatosis model remains largely unknown. Therefore, we hypothesized that iron supplementation, a common and effective approach to treating iron deficiency^14^, could prevent the progression of obesity and hepatic steatosis and designed the present study to test this hypothesis. This study provides insights into the effect of iron supplementation on mitochondrial-related energy metabolism signaling pathways through comprehensive gene expression analysis in the liver and skeletal muscle of iron-supplemented high-fat diet-induced obese mice, addressing the gap to date.

## Results

### Iron supplementation attenuates the increase in body weight and plasma lipids

**Figure 1:**
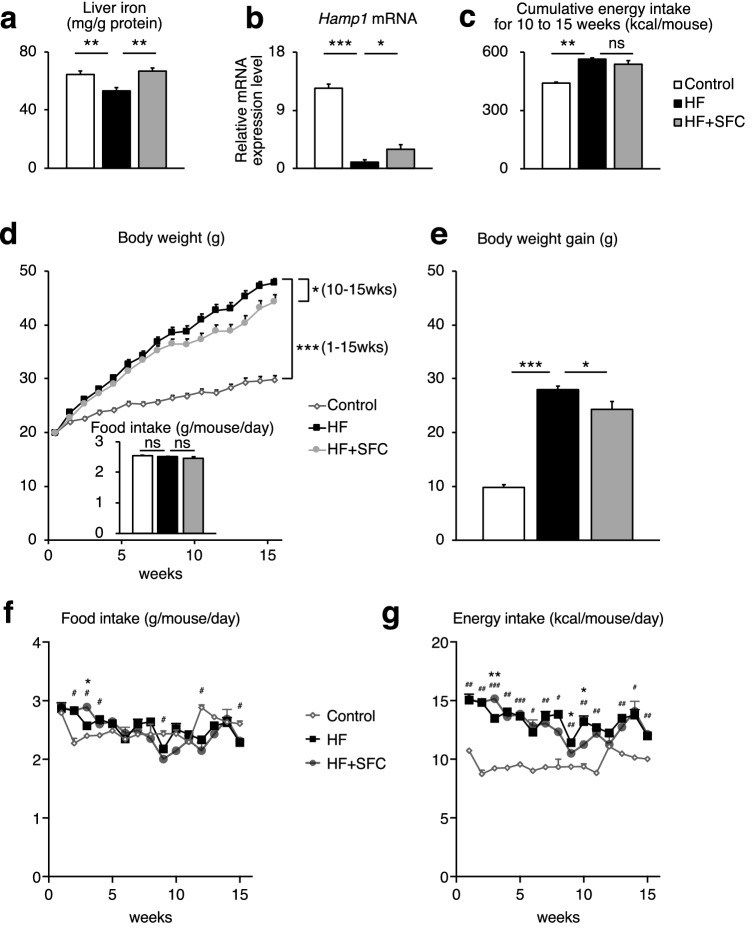
Iron supplementation reduces diet-induced weight gain. (**a**–**g**) Male C57BL/6J mice of 6 weeks of age were fed with an open source control diet (Control; 10% kcal fat), a high-fat diet (HF; 60% kcal fat), or a high-fat diet supplemented with sodium ferrous citrate (HF + SFC) for 15 weeks. (**a**) Liver iron content. The values were corrected using tissue protein levels (*n* = 5–6). (**b**) Analysis of *Hamp1* mRNA expression (*n* = 4–6). (**c**) Cumulative energy intake for 10–15 weeks per mouse per cage. (**d**) Body weight and food intake in the different groups (*n* = 11–12). Food intake was measured for 15 weeks. (**e**) Body weight gain from 0 to 15 weeks after administering (*n* = 11–12). (**f**, **g**) Longitudinal, (**f**) food, or (**g**) energy intake. Data are shown as mean ± SEM values. Statistical analysis was performed using one-way ANOVA followed by Holm–Sidak’s post hoc test. (**a**–**e**) *p < 0.05; **p < 0.01; ***p < 0.001; ns, not significant. (Significant differences vs. HF). (**f**, **g**) *p < 0.05. (Significant differences in HF vs. HF + SFC). ^#^*p* < 0.05; ^##^*p* < 0.01; ^###^*p* < 0.001. (Significant differences in HF vs. Control).

**Figure 2:**
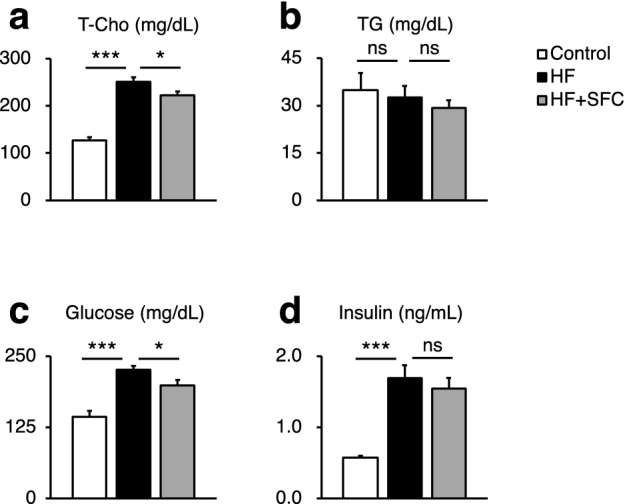
Metabolic parameters. (**a**–**d**) Male C57BL/6 J mice of 6 weeks of age were fed with each diet for 15 weeks as specified in Fig. 1. (**a**, **b**) Plasma levels of (**a**) total cholesterol (T-Cho); and (**b**) triglycerides (TG) in C57BL/6J mice after 15 weeks on different diets (*n* = 5–6). (**c**, **d**) (**c**) Blood glucose; and (**d**) plasma insulin levels after 8 weeks on different diets (*n* = 12). Data are expressed as mean ± SEM values. Statistical analysis was performed using one-way ANOVA followed by Holm–Sidak’s post hoc test. *p < 0.05; ***p < 0.001; ns, not significant. (Significant differences vs. HF).

We first evaluated the metabolic effects of iron supplementation using sodium ferrous citrate (SFC) in male C57BL/6J mice, which received either an open source control diet (Control), high-fat diet (HF), or HF supplemented with SFC (HF + SFC) for 15 weeks. Since the liver is a major iron storage organ and reflects changes in iron storage associated with dietary iron supplementation^15^, we measured hepatic iron level as an index of body iron. The findings showed that the hepatic iron level was significantly decreased in HF-fed mice compared to Control-fed mice (Fig. 1a). Hepcidin is a master hormone regulator of iron metabolism and is produced little or none when intracellular iron is deficient. The expression of *Hamp1*, which encodes hepcidin, was also significantly decreased in the liver (Fig. 1b). These results are common symptoms of iron deficiency induced by HF feeding^16^. Compared to HF-fed mice, iron supplementation significantly increased hepatic iron level and *Hamp1* gene expression (Fig. 1a,b), indicating that HF-induced iron deficiency is attenuated by iron supplementation. Compared with Control-fed mice, the body weight of HF-fed mice increased significantly 1 week following diet initiation (Fig. 1d). The total body weight gain was also significantly increased (Fig. 1e). However, compared to HF-fed mice, the increase in body weight of iron supplemented mice was significantly suppressed from 10 to 15 weeks, and total body weight gain was significantly decreased despite no changes in average food and cumulative energy intake (Fig. 1c–g). The weight of epididymal white adipose tissue (epiWAT) and mesenteric white adipose tissue (mWAT) was significantly increased in HF-fed mice, compared with that of Control-fed mice (see Supplementary Fig. S1a,b). In addition, the mWAT weight was 1.85 ± 0.17 g in HF-fed mice and 1.34 ± 0.21 g in HF + SFC-fed mice (*p* = 0.058, HF vs. HF + SFC) (see Supplementary Fig. S1b). Furthermore, the body temperature of the HF-fed mice was significantly lower than that of the Control-fed mice (see Supplementary Fig. S1c). The body temperature was 36.93 ± 0.07 ℃ in HF-fed mice and 37.10 ± 0.07 ℃ in HF + SFC-fed mice (*p* = 0.095, HF vs. HF + SFC). Plasma total cholesterol (T-Cho), insulin, and blood glucose levels were significantly increased in HF-fed mice compared with those of the Control-fed mice (Fig. 2a,c,d). Compared with HF-fed mice, iron supplementation significantly reduced T-Cho levels and glucose levels (Fig. 2a,c), whereas plasma triglycerides (TG) and insulin levels did not show significant changes by iron supplementation (Fig. 2b,d).

### Iron supplementation reduces hepatic lipid accumulation

**Figure 3:**
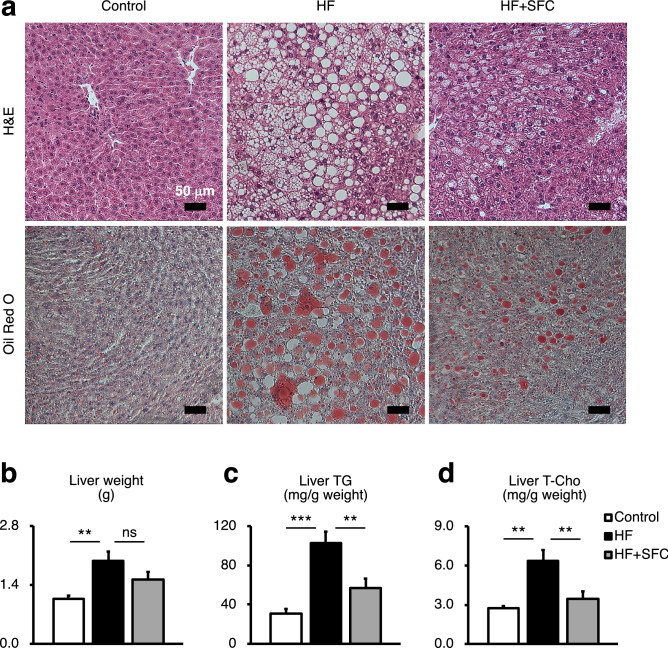
Iron supplementation reduces hepatic lipid accumulation. (**a**–**d**) Male C57BL/6J mice of 6 weeks of age were fed with each diet for 15 weeks as specified in Fig. 1. (**a**) Representative image of Hematoxylin–eosin (H&E) and Oil Red O stained liver sections. Scale bar is 50 μm. (**b**) Weight of liver (*n* = 5–6). (**c**, **d**) (**c**) Liver triglycerides (TG); and (**d**) total cholesterol (T-Cho) content (*n* = 5–6). The values were corrected by the tissue weight. Data are expressed as mean ± SEM values. Statistical analysis was performed using one-way ANOVA followed by Holm–Sidak’s post hoc test. **p < 0.01; ***p < 0.001; ns, not significant. (Significant differences vs. HF).

The results of Hematoxylin–Eosin (H&E) staining and Oil Red O staining consistently showed severe lipid accumulation in the livers of HF-fed mice compared to that of the Control-fed mice (Fig. 3a). However, the accumulation of lipid droplets in the liver of mice supplemented with iron was less than that of mice fed with HF (Fig. 3a). Furthermore, H&E staining would suggest not only a change in total liver lipid, but also the morphology of the lipid droplets. The size of these lipid droplets was smaller in iron-supplemented mice than that in HF-fed mice. Consistent with the above histological findings, HF-fed mice showed significantly increased liver weight, TG, and T-Cho levels compared with those of Control-fed mice, whereas iron supplementation significantly reduced TG and T-Cho accumulation compared with those of HF-fed mice (Fig. 3b-d).

### Iron supplementation reduces morphological abnormalities of the mitochondria and increases transcription of genes associated with energy metabolism in the liver and skeletal muscle

**Figure 4:**
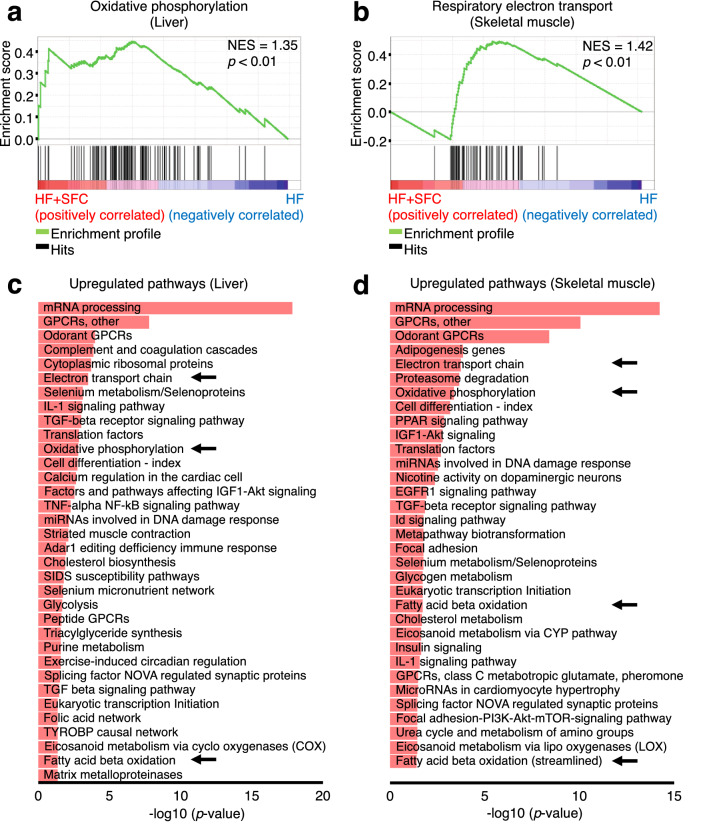

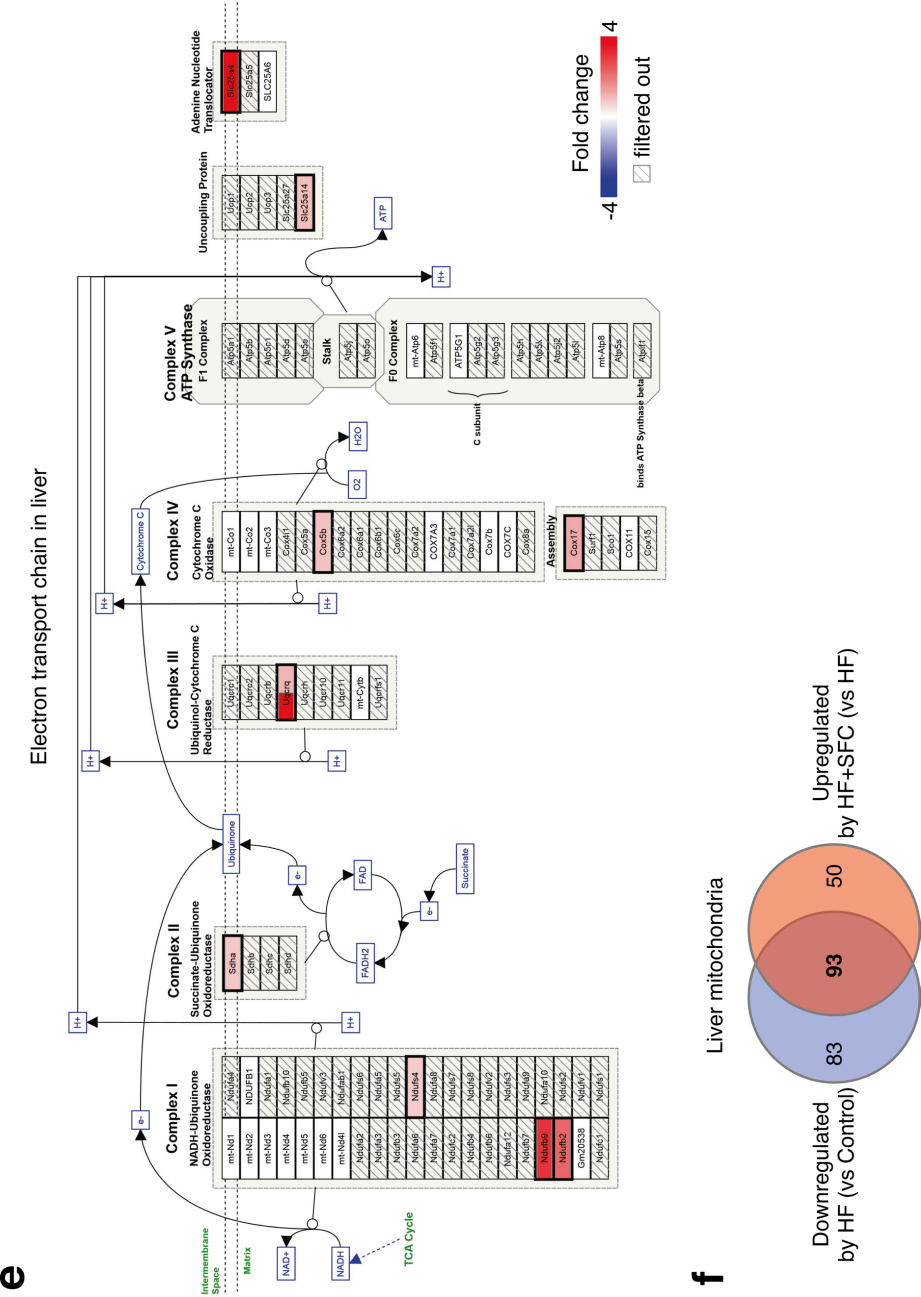

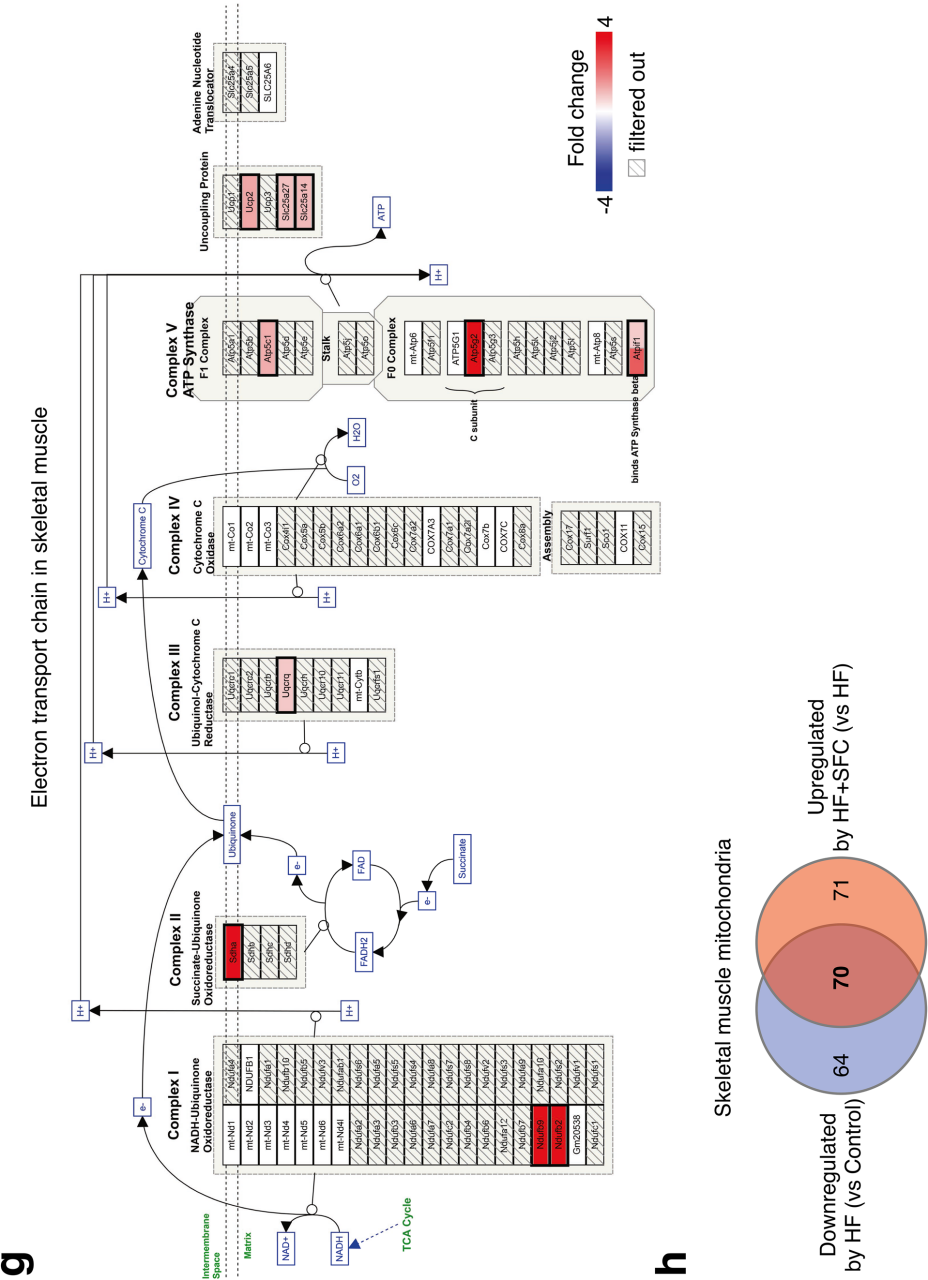
Microarray analysis shows iron supplementation improves transcription of energy metabolism and mitochondrial related genes in the liver and skeletal muscle. (**a**–**h**) Male C57BL/6J mice of 6 weeks of age were fed with each diet for 15 weeks as specified in Fig. 1. For each group, a mix of all sample cDNAs was used. (**a**, **b**) Enrichment plots of genes upregulated in HF + SFC (**a**) liver; and (**b**) skeletal muscle (compared to HF). (**a**) The Kyoto Encyclopedia of Genes and Genomes (KEGG) database; or (**b**) Reactome database was used. The analysis was performed by Gene Set Enrichment Analysis (GSEA). *NES* normalized enrichment score. (**c**, **d**) Pathways containing upregulated genes by greater than or equal to 1.5-fold in HF + SFC (**c**) liver; and (**d**) skeletal muscle (compared to HF) (*p* < 0.05). WikiPathways database was used. The analysis was performed by Transcriptome Analysis Console (TAC). (**e**, **g**) Pathway mapping for electron transport chain (ETC) in (**e**) liver; and (**g**) skeletal muscle. Upregulated genes in HF + SFC are shown as red gradations (compared to HF). The analysis was performed by TAC. The pathway mapping was created on the basis of the Wikipathways database (https://www.wikipathways.org/index.php/Pathway:WP295). (**f**, **h**) Venn diagrams representing the number of differentially expressed mitochondrial genes (greater than or equal to 1.5-fold) in (**f**) liver; and (**h**) skeletal muscle. Mitochondrial genes were based on the MitoCarta2.0 database.

To explore the molecular basis of iron-induced reduction in body weight gain and hepatic lipid accumulation, we investigated the transcription of genes associated with energy metabolism in the liver and skeletal muscle in HF-fed and HF + SFC-fed groups. Gene Set Enrichment Analysis (GSEA) indicated that genes upregulated by iron supplementation were highly enriched in the mitochondrial-associated signaling pathways that regulate processes such as oxidative phosphorylation, respiratory electron transport or translation in the liver and skeletal muscle (Fig. 4a,b, and Supplementary Fig. S2a). Similarly, as indicated by the arrows, an analysis using Transcriptome Analysis Console (TAC) software identified the electron transport chain (ETC), oxidative phosphorylation, and fatty acid beta-oxidation as pathways containing genes upregulated by iron supplementation in both the liver and skeletal muscle (Fig. 4c,d). In the mitochondrial ETC pathway of the liver and skeletal muscle, highly upregulated genes by iron supplementation included those encoding each respiratory chain complex I–V, uncoupling of proteins and/or adenine nucleotide translocator (Fig. 4e,g). Furthermore, HF + SFC-fed group upregulated more than half of the downregulated mitochondrial genes in the HF-fed group (93 genes in the liver, 52.84%; 70 genes in the skeletal muscle, 52.24%) (Fig. 4f,h); HF-induced mitochondrial abnormalities may have been prevented by iron supplementation. These data suggest that mitochondria may play an important role in mechanisms via which iron supplementation attenuates a decline in energy metabolism in the HF-fed group.

**Figure 5:**
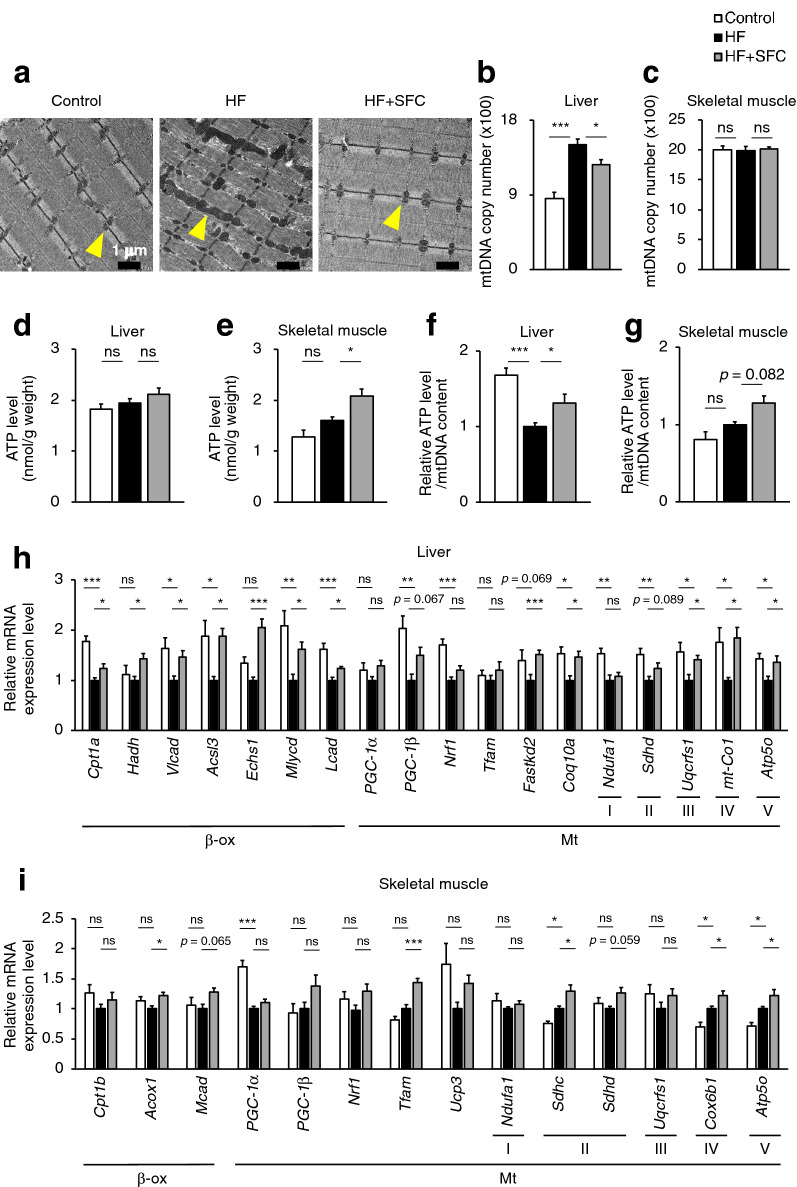
Iron supplementation reduces mitochondrial morphological abnormalities and increases mitochondrial-associated gene expression. (**a**–**i**) Male C57BL/6J mice of 6 weeks of age were fed with each diet for 15 weeks as specified in Fig. 1. (**a**) Representative image of transmission electron microscopy analysis in skeletal muscle. Yellow arrows point to the mitochondria. Scale bar is 1 μm. (**b**, **c**) Copy number of mitochondrial DNA (mtDNA) in (**b**) liver and (**c**) skeletal muscle (*n* = 5–6). (**d**, **e**) ATP levels normalized by tissue weight in (**d**) liver and (**e**) skeletal muscle (*n* = 5–6). (**f**, **g**) ATP levels normalized by mtDNA in (**f**) liver and (**g**) skeletal muscle (*n* = 5–6). (**h**, **i**) Analysis of mRNA expression related to fatty acid beta oxidation (β-ox), mitochondrial (Mt), mitochondrial respiratory chain complex I (I), II (II), III (III), IV (IV), and V (V) in (**h**) liver; and (**i**) skeletal muscle (*n* = 5–6). Data are expresses as mean ± SEM values. Statistical analysis was performed using a one-way ANOVA followed by Holm–Sidak’s post hoc test. *p < 0.05; **p < 0.01; ***p < 0.001; ns, not significant. (Significant differences vs. HF).

As shown in Fig. 5a, the morphometric analysis of skeletal muscle via electron microscopy revealed regularly aligned small mitochondria in Control-fed mice. However, the mitochondria were frequently fused or swollen and the organization of sarcomeres was disturbed in HF-fed mice. These morphological abnormalities of mitochondria are observed in aged rodents’ skeletal muscle with declined energy metabolism^17^. In contrast, well organized and regularly arranged sarcomere and mitochondria were observed in the skeletal muscle of iron supplemented mice, indicating the attenuating effects of iron on the morphological abnormalities induced by HF feeding.

In the liver, the copy number of mitochondrial DNA (mtDNA) was significantly higher in HF-fed mice than that in Control-fed mice, and significantly decreased in HF + SFC-fed mice compared to that in HF-fed mice (Fig. 5b). In contrast, in skeletal muscle, there were no significant changes among the three groups (Fig. 5c). ATP levels in the liver were not significantly different among the three groups (Fig. 5d). In the skeletal muscle, however, they were significantly increased in HF + SFC-fed mice compared to that in HF-fed mice (Fig. 5e). Furthermore, in the liver of HF-fed mice, the ATP levels normalized by mtDNA (ATP/mtDNA) were significantly decreased compared to those in Control-fed mice (Fig. 5f). In contrast, iron supplementation significantly increased hepatic ATP/mtDNA in HF + SFC-fed mice than in HF-fed mice (Fig. 5f). The ATP/mtDNA of skeletal muscle were 1.00 ± 0.04 in HF-fed mice and 1.28 ± 0.09 in HF + SFC-fed mice (*p* = 0.082, HF vs. HF + SFC) (Fig. 5g).

Next, we evaluated the expression of the genes regulating energy metabolism using qRT-PCR (Fig. 5h,i). In the liver, expression of genes involved in beta-oxidation and mitochondrial ETC were significantly decreased in HF-fed mice compared to the Control-fed mice. On the contrary, in the skeletal muscle, the expression of the genes encoding the mitochondrial ETC were significantly increased in HF-fed mice. Previous studies have shown that mitochondrial ETC levels are decreased in the liver but increased in the skeletal muscle by high fat diet feeding^18,19^, showing a trend similar to our data. Furthermore, in the liver, comparing HF-fed mice with HF + SFC-fed mice, iron supplementation significantly increased the expression of genes encoding components of beta-oxidation, such as carnitine palmitoyltransferase 1a (*Cpt1a*), hydroxyacyl-CoA dehydrogenase (*Hadh*), very long-chain acyl-CoA dehydrogenase (*Vlcad*), acyl-CoA synthetase long-chain family member 3 (*Acsl3*), enoyl-CoA hydratase short chain 1 (*Echs1*), malonyl-CoA decarboxylase (*Mlycd*), and long-chain acyl-CoA dehydrogenase (*Lcad*). In addition, the expression of genes involved in maintaining the function of the mitochondrial ETC, such as FAST kinase domains 2 (*Fastkd2*) and coenzyme Q10A (*Coq10a*), and those encoding mitochondrial ETC components, such as ubiquinol-cytochrome c reductase Rieske iron–sulfur polypeptide 1 (*Uqcrfs1*), mitochondrially encoded cytochrome c oxidase I (*mt-Co1*), and the ATP synthase H+ transporting mitochondrial F1 complex O subunit (*Atp5o*), were significantly increased by iron supplementation (Fig. 5h). In the skeletal muscle, similarly, the expression of genes encoding components of beta oxidation and the mitochondrial ETC, such as acyl-CoA oxidase 1 (*Acox1*), succinate dehydrogenase complex subunit C (*Sdhc*), cytochrome c oxidase subunit 6B1 (*Cox6b1*), and *Atp5o* were significantly increased by iron supplementation. The expression of other mitochondrial related gene such as mitochondrial transcription factor A (*Tfam*) was also significantly increased (Fig. 5i). It should be considered that *Acox1*-encoded peroxisomal oxidation is very limited in skeletal muscle, and the functional role and importance of uncoupling protein 3 (*Ucp3*) is still very much up for debate. However, these findings supported the results of comprehensive gene expression analyses (Fig. 4).

### Iron supplementation upregulates the synthetic pathways of heme and iron–sulfur (Fe–S) cluster, the iron prosthetic groups, in the liver and skeletal muscle

**Figure 6:**
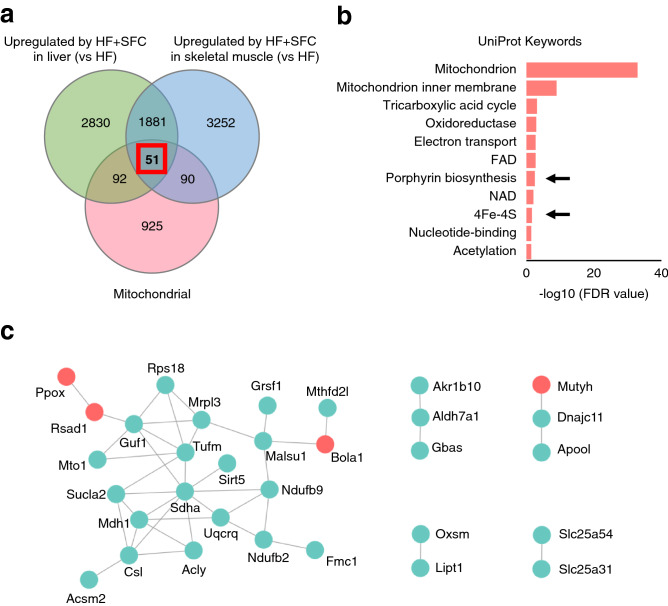
Iron supplementation alters the transcription of mitochondrial genes associated with the iron prosthetic groups in the liver and skeletal muscle. (**a**–**c**) Male C57BL/6J mice of 6 weeks of age were fed with each diet for 15 weeks as specified in Fig. 1. For each group, a mix of all sample cDNAs was used. (**a**) Venn diagrams showing upregulated expression of 51 mitochondrial genes in the liver and skeletal muscle of HF + SFC compared with HF (greater than or equal to 1.5-fold). Mitochondrial genes were based on the MitoCarta2.0 database. (**b**) STRING enrichment analysis of 51 upregulated mitochondrial genes in the liver and skeletal muscle of HF + SFC revealed in (**a**) (FDR < 0.05). Arrows point to the iron prosthetic groups related pathways. UniProt database was used. The analysis was performed by Cytoscape. (**c**) The STRING networks of 51 upregulated mitochondrial genes in the liver and skeletal muscle of HF + SFC revealed in (**a**). These networks are annotated with energy generation and ETC using BiNGO (*p* < 0.001). The iron prosthetic group related genes (red) and other genes (green) are shown. The analysis was performed by Cytoscape.

To investigate the mechanism by which iron supplementation increased the transcription of mitochondrial-associated genes, we focused on 51 common mitochondrial genes whose expression levels were upregulated in both liver and skeletal muscle of the HF + SFC-fed group compared to the HF-fed group (Fig. 6a, Supplementary Table S1). These genes were highly enriched in pathways associated with the heme and iron–sulfur (Fe–S) cluster, in addition to the mitochondrial energy metabolism (Fig. 6b). Heme and Fe–S cluster are iron prosthetic groups formed from iron processed in mitochondria and contained in ETC complex subunits. Six (11.76%) of the 51 genes were annotated with heme and Fe–S cluster, four genes formed energy generation and ETC associated networks with other mitochondrial genes (Fig. 6c), suggesting the involvement of heme- and Fe–S cluster-associated mitochondrial genes in mitochondrial energy metabolism in the liver and skeletal muscle of HF + SFC-fed mice.

**Figure 7:**
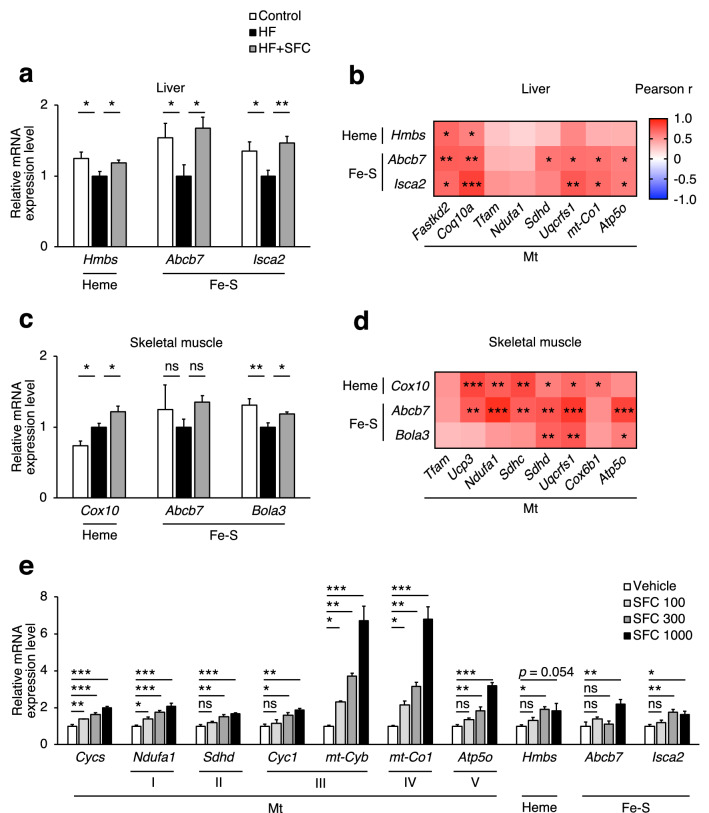
Iron supplementation upregulates the gene expression of iron prosthetic groups synthesis. (**a**–**d**) Male C57BL/6J mice of 6 weeks of age were fed with each diet for 15 weeks as specified in Fig. 1. (**a**, **c**) mRNA expression analysis related to the synthesis of heme (Heme) and Fe–S cluster (Fe–S) in (**a**) liver; and (**c**) skeletal muscle (*n* = 5–6). Data are expressed as mean ± SEM values. Statistical analysis was performed using a one-way ANOVA followed by Holm–Sidak’s post hoc test. *p < 0.05; **p < 0.01; *ns* not significant. (Significant differences vs. HF). (**b**, **d**) Correlation heatmap between relative mRNA expression levels of mitochondrial (Mt) related genes and Heme or Fe–S related genes in the (**b**) liver; and (**d**) skeletal muscle of HF and HF + SFC (*n* = 12). Data were analysed by Pearson correlation coefficient. *p < 0.05; **p < 0.01; ***p < 0.001. (**e**) Analysis of mRNA expression related to Mt, Heme, and Fe–S from primary hepatocytes isolated from 5-weeks-old male C57BL/6J mice cultured for 24 h (*n* = 4). These assay mediums were either supplemented with SFC 100 μM (SFC 100), SFC 300 μM (SFC 300), SFC 1000 μM (SFC 1000), or did not contain any supplements (Vehicle). All media contains 0.05% DMSO. Data are expressed as mean ± SEM values. Statistical analysis was performed via one-way ANOVA followed by Holm-Sidak’s post hoc test. *p < 0.05; **p < 0.01; ***p < 0.001; *ns* not significant. (Significant differences vs. Vehicle).

Furthermore, three genes in the main network—*Ppox, Bola1*, and *Rsad1*—are involved in heme or Fe–S clusters synthesis (Fig. 6c). Similarly, the heme skeleton porphyrin synthesis pathway was also upregulated with iron supplementation (Fig. 6b, Supplementary Fig. S2b). The results of qRT-PCR showed that iron supplementation significantly increased the expression of genes involved in the heme synthesis pathway, such as hydroxymethylbilane synthase (*Hmbs*) and farnesyltransferase cytochrome c oxidase assembly factor 10 (*Cox10*) in the liver and skeletal muscle compared with HF-fed mice (Fig. 7a,c). In addition, expression of genes involved in the Fe–S cluster assembly synthesis pathway, such as ATP-binding cassette sub-family B member 7 (*Abcb7*), iron–sulfur cluster assembly 2 homolog mitochondrial (*Isca2*), and/or bolA-like 3 (*Bola3*), were significantly increased by iron supplementation (Fig. 7a,c). Moreover, the mRNA expression levels of these genes involved in heme or Fe–S cluster synthesis were positively correlated with those involved in mitochondria and their ETC (Fig. 7b,d).

Furthermore, stimulation of mouse primary hepatocyte by iron triggered the expression of genes associated with mitochondrial ETC, heme synthesis and Fe–S cluster synthesis in a dose-dependent manner (Fig. 7e). These results suggest that iron supplementation may have increased iron prosthetic groups contained in the mitochondrial electron transport system, thereby improving mitochondrial function.

## Discussion

**Figure 8:**
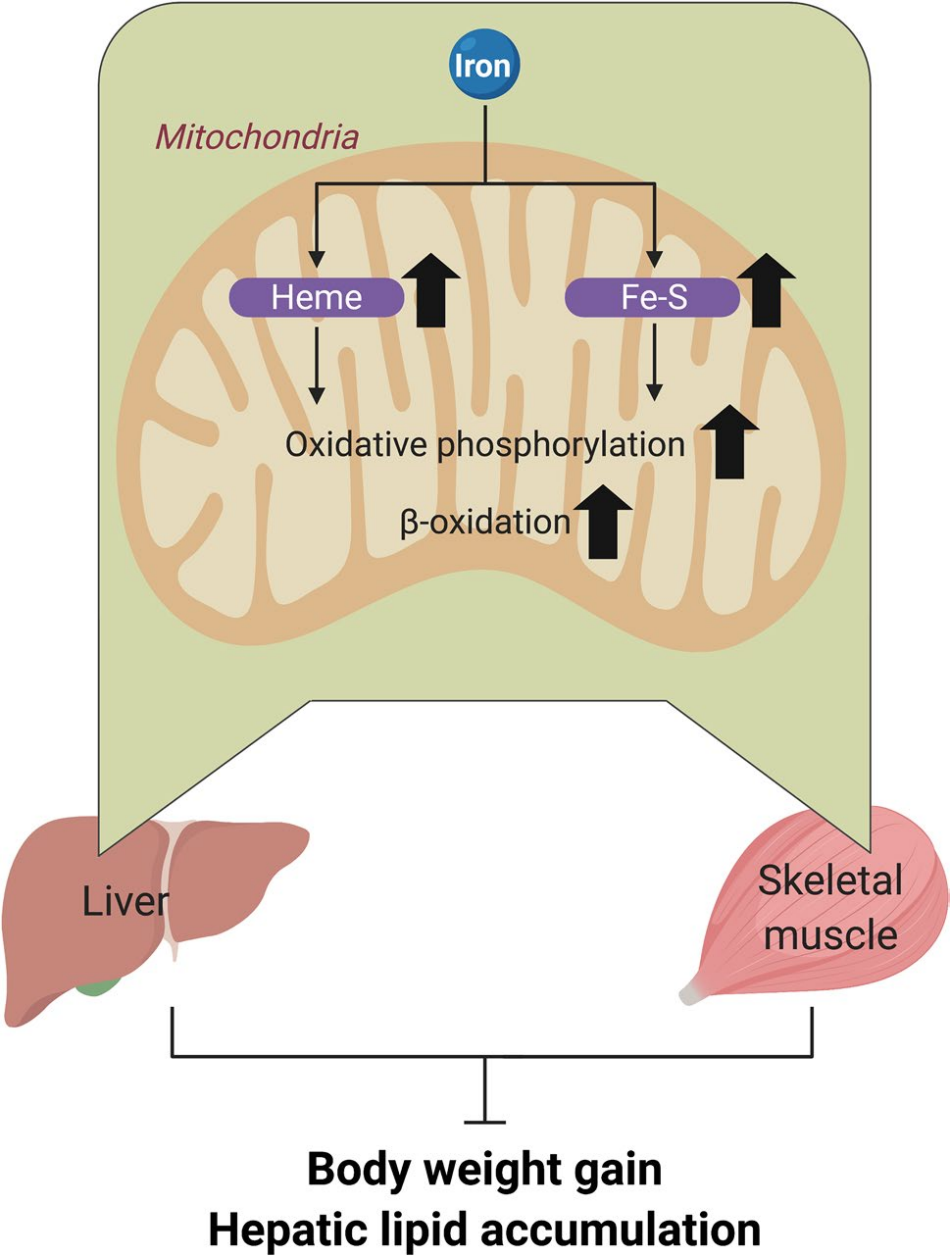
Suggested mechanism for the reduction in diet-induced weight gain and hepatic lipid accumulation by iron supplementation.

The current study administered iron to diet-induced obese mouse models to investigate the effect of iron supplementation on obesity and hepatic steatosis. We found that diet-induced weight gain and hepatic lipid accumulation were reduced by iron supplementation. Furthermore, we found that iron regulates mitochondrial signaling pathways—gene transcription of mitochondrial component molecule synthesis and their energy metabolism. Iron supplementation reduced morphological abnormalities of the mitochondria in skeletal muscle and increased the expression of genes related to mitochondrial ETC and energy metabolism in skeletal muscle and liver. The mechanism underlying these effects may be associated with the synthesis of iron prosthetic groups contained in the mitochondrial ETC (Fig. 8).

The liver, a major site for iron storage and lipid metabolism, plays a vital role in the interaction and control of these two metabolic pathways^20^. Previous studies have demonstrated that iron distribution in tissues is altered by obesity, and that hepatic iron storage is reduced in mice fed a high-fat diet^16,21,22^. In accordance, we also observed hepatic iron deficiency in HF-fed mice (Fig. 1a). Furthermore, it has also been shown that iron deficiency is associated with the progression of steatosis and cellular TG accumulation in the liver^7,23–25^. These symptoms are consistent with the liver phenotype of hepatic iron-deficient obese mice in our experiment (Fig. 3). These findings suggest that hepatic iron deficiency accompanying obesity may aggravate hepatic lipid metabolism abnormalities and steatosis. In addition, the present study revealed that iron supplementation reduced the increase in plasma and hepatic lipids (Figs. 2, 3). Overall, the new findings of the present study show that iron may regulate the liver lipid metabolism and that hepatic steatosis accompanied by iron deficiency may be reduced by iron supplementation.

Most iron in the mammalian cells is directed to heme or Fe–S cluster cofactor synthesis^26,27^, and mitochondria, a primary site for the synthesis of both, plays an important role in their regulation^27^. Many heme- and Fe–S cluster-containing proteins essential for ATP synthesis, such as ETC complex subunits and cytochromes, are present in mitochondria. Heme is required for complexes II, III, and IV to function properly^28^, while Fe–S clusters are contained in complexes I, II, and III^27^.

Accumulated evidence indicates that heme and Fe–S cluster synthesis pathways play an important role in maintaining mitochondria. Both heme levels and mitochondrial cytochrome c oxidase activity decrease with aging^29,30^. Moreover, deficiency of 5-aminolevulinate synthase (ALAS) 1, a rate-limiting factor of heme synthesis, induces diminished mitochondrial function in aged mice^31^. Similarly, mutations in other genes encoding heme synthesis are associated with mitochondrial diseases such as cytochrome c oxygenase deficiency^32^. Similar to the effect of mutations in the heme synthesis gene, mutations in genes involved in Fe–S cluster synthesis are implicated in mitochondrial diseases, such as the multiple mitochondrial dysfunction syndrome (MMDS)^33,34^, early onset of severe autosomal recessive disease causing various abnormal neurodevelopmental, lactic acidosis, and premature death.

Thus, the depletion of heme and Fe–S proteins may reduce the activity of the mitochondrial respiratory chain complex, causing mitochondrial dysfunction^29,35,36^. Interestingly, in this study, the expression of genes involved in both heme and Fe–S cluster synthesis pathways decreased in skeletal muscle and liver of HF-fed mice, compared with that of Control-fed mice (data set have been submitted at NCBI's Gene Expression Omnibus GSE161644 and GSE161646). Therefore, the downregulation of mitochondrial heme and Fe–S cluster synthesis pathways may contribute to reduced mitochondrial function in high-fat diet-induced obesity sufferers.

Iron supplementation significantly elevated the expression of *Cox10*, the mutations of which are associated with mitochondrial disease, and *Bola3* and *Isca2*, the mutations of which are associated with MMDS and/or oxidative phosphorylation activity^33,37,38^ (Fig. 7a,c). These results indicate that upregulation of iron prosthetic group synthesis via iron supplementation may be central to improving mitochondrial function.

Comparing HF-fed mice and HF + SFC-fed mice, there was no difference in the intake of diet components except for iron (see Supplementary Tables S2 and S3 online). However, SFC contains ferrous iron (Fe^2+^), and the HF contains ferric iron (Fe^3+^), suggesting that different iron states may be a factor influencing iron absorption. Duodenal cytochrome B (*Dcytb*) reduces Fe^3+^ in HF to Fe^2+^, enabling effective iron absorption in the duodenum^39^, and it has been shown that high-fat diet feeding decreases duodenal *Dcytb* expression and thereby may contribute to iron deficiency^40^. In contrast, Fe^2+^ in SFC might not require the reduction by *Dcytb* and could be directly absorbed by divalent metal transporter 1 (*Dmt1*), which plays a major role in iron absorption^41^. Though it is difficult to conclude clearly from the results of this study, it suggests that the absorption of iron in SFC might not be inhibited by HF feeding.

Our study has a few limitations. First, absence of a Control + SFC group—Our study focused on clarifying the effects of iron with the diet-induced obesity model; it is difficult to establish whether there are effects of iron supplementation in the absence of HF feeding. Therefore, the effects of iron supplementation on other diets need to be carefully discussed.

Second, mice were housed by groups rather than individually, which is a weakness in the assessment of food and energy intake.

Third, the percentage of sucrose in the diet was different between the Control diet (35 kcal%) and HF diet (7 kcal%). Therefore, differences in sucrose intake may affect metabolism.

Fourth, HF-fed mice had significantly higher caloric intake than Control-fed mice (Fig. 1c), indicating that HF-fed mice ate more micronutrients than the Control-fed mice. Interestingly, HF-fed mice caused iron deficiency in the liver, despite higher intake of iron compared to Control-fed mice (Fig. 1a). However, the detailed mechanism, which is probably related to absorption and circulation, is unknown from our results.

Regarding liver effects, previous studies have indicated that iron deficiency in the liver may be attributed to obesity and hepatic steatosis, but iron overload may be responsible for the deterioration of NASH, which is caused by the progression of hepatic steatosis^42–44^. In the liver, iron overload may induce oxidative stress by producing free radicals, stimulate hepatic stellate cells, and increase collagen production, which can accelerate the progression of liver fibrosis^45^. In this study, the mouse diet-induced obesity models neither exhibited severe fibrosis nor reached NASH. Therefore, iron supplementation was effective, whereas iron supplementation in the NASH model could aggravate the oxidative stress and contribute to the development of NASH. Therefore, to put iron supplementation into practical use to improve energy metabolism and hepatic steatosis, it is important to consider whether iron supplementation will be effective or lead to deterioration, as observed in the NASH model. In the future, in addition to investigating the obesity model, an in-depth investigation into the effect of iron on the NASH model is required.

The above results indicate that the effect of iron may change according to internal conditions, such as the presence or absence of diseases, including obesity. Under conditions of functional iron deficiency, accompanied by impaired erythropoiesis and haemoglobin synthesis, normal or high dietary iron supplementation may cause pathological iron accumulation in tissues^46^. This suggests that iron supplementation may be beneficial or detrimental depending on physical conditions. Therefore, when supplementing patients with iron, it is prudent to priorly evaluate the patient’s iron accumulation levels and administer the appropriate amount of iron supplements. However, complications of iron deficiency and chronic diseases make it difficult to diagnose iron status clearly^47^. Serum ferritin, serum iron, total iron-binding capacity (TIBC), and transferrin levels, the common biomarkers for iron deficiency, fluctuate with an acute and chronic infection or inflammation^47^. Therefore, the development of biomarkers beneficial for iron deficiency with chronic disease complications, including obesity, should be explored. Several current therapeutic strategies for chronic diseases are aimed at targeting muscle mitochondria to increase energy metabolism efficiency. It has been suggested that supplementing optimal iron may be important for an appropriate response to such attempts, and particularly in T2DM, the need for detailed studies on iron substitution has been discussed^48^. Our study shows that iron supplementation could be an effective strategy to prevent the progression of obesity and hepatic steatosis, which may serve to advance studies on iron and energy metabolism, for which many unclear mechanisms remain.

## Methods

### Animal experiments

This study was approved by the institutional review board at Keio University. All experiments were performed in accordance with the Keio University animal experimentation guidelines. All animal studies were carried out in compliance with the ARRIVE guidelines. Five-week-old male C57BL/6J mice (Japan SLC, Inc., Shizuoka, Japan) were acclimated for 1 week. At 6 weeks of age, their diets were changed to an open source control or HF diet with or without iron supplementation. Body weight and food intake were measured weekly. The open source control diet (D12450B) contained 10 kcal% fat, 20 kcal% protein, and 70 kcal% carbohydrates, whereas the HF diet (D12492) contained 60 kcal% fat, 20 kcal% protein, and 20 kcal% carbohydrates (Research Diets, Inc., New Brunswick, NJ, USA). Both the open source control diet and HF diet originally contained iron (see Supplementary Table S3 online). Iron supplemented mice were fed with 0.023% (w/w) SFC (Komatsuya, Osaka, Japan) simultaneously as the HF diet. These food compositions are shown in the Supplementary Tables S2 and S3 online. Mice always had free access to food and water. After 7 weeks of each diet, body temperature of the rectum was measured using a thermo-register for mice (AD-1687) (A&D, Tokyo, Japan). After 8 weeks of each diet, glucose parameters were measured. After 15 weeks of each diet, mice were fasted for 4 h and anesthetized with isoflurane, and tissues were collected. Each tissue was weighed and then rapidly frozen in liquid nitrogen. It was then used for RNA isolation, lipid, iron, and ATP measurements, and histological analysis. The facility where all the mice were housed had a 12-h light (from 8 a.m. to 8 p.m.)-dark (from 8 p.m. to 8 a.m.) cycle and temperature control at 23–25 ℃.

### Biochemistry and determination of glucose and lipids

After 8 weeks of each treatment, blood and plasma were collected from the tail of mice fasted for 6 h from 8 a.m. to 2 p.m. to measure glucose and insulin. Blood glucose was measured using Life check (GUNZE Limited, Osaka, Japan). Blood was heparinized, and plasma insulin concentrations were measured using ELISA (Morinaga Institute of Biological Science, Inc., Kanagawa, Japan). After 15 weeks of each treatment, plasma was collected from the vena cava of mice fasted for 4 h from 6 a.m. to 10 a.m. and lipids were measured. Plasma triglycerides (TG) and total cholesterol (T-Cho) were measured using commercial enzymatic assay kits. Enzymatic assay kits used were as follows: TG—Determiner L (Kyowa Medex, Tokyo, Japan); and T-Cho—Cholesterol E-test Wako (Wako, Osaka, Japan). Liver lipid measurements were performed as described previously^49^. Briefly, lipids were extracted from the liver using the Folch method^50^. Approximately 50 mg of the center of each tissue sample was cut out from samples stored at − 80 °C. Each tissue sample was homogenized in methanol at room temperature. The same amount of chloroform (as methanol) was added to the extract. The extract was washed with ultrapure water and centrifuged at 15,000 rpm. The lower layer was transferred to a new tube and dried for one week to evaporate the chloroform. The dried product was resuspended in isopropanol and used for lipid measurements. As in the measurement of plasma lipids, liver TG was measured using Determiner L (Kyowa Medex, Tokyo, Japan), and liver T-Cho was measured using the Cholesterol E-test Wako (Wako, Osaka, Japan). Multiskan FC (Thermo Fisher Scientific, Waltham, MA, USA) was used for data measurement.

### mRNA expression analysis by qRT-PCR

RNeasy Fibrous Tissue Mini Kit (Qiagen, Hilden, Germany) was used to extract skeletal muscle total RNA, and RNeasy Mini Kit (Qiagen, Hilden, Germany) was used for other tissues. Total RNA was extracted from the tissue cryopreserved with liquid nitrogen according to the kit’s instructions. Next, cDNA was synthesised according to the instructions using a kit of PrimeScript RT Master Mix (TaKaRa, Siga, Japan). Gene expression was measured by real-time PCR using synthesised cDNA. SYBR Premix Ex Taq II (TaKaRa, Siga, Japan) and THUNDERBIRD SYBR qPCR Mix (Toyobo, Osaka, Japan) were used according to the kit’s instructions. Measurements were performed via PikoReal and QuantStudio 5 (Thermo Fisher Scientific, Waltham, MA, USA). The primer set sequences are listed in the Supplementary Table S4 online.

### Microarray analysis

Liver and skeletal muscle total RNA and cDNA were prepared as described above. The microarray was performed on an Affymetrix GeneChip Mouse Genome 430 2.0 Array, and data analysis was performed using TAC Software (Thermo Fisher Scientific, Waltham, MA, USA), version 4.0, GSEA, version 3.0, Venny, version 2.1.0, and Cytoscape, version 3.8.0. TAC analysis used the WikiPathways database, and GSEA analysis used MSigDB, version 6.2 and 7.2. Cytoscape analysis used UniProt database and stringApp, version 1.5.1 for enrichment analysis; and Gene Ontology database and BiNGO, version 3.0.4 for network annotation. Mitochondrial genes were based on MitoCarta2.0.

### Measurement of iron levels

Intracellular iron concentration was measured using Metallo Assay Kit (Metallogenics, Chiba, Japan). To measure the intracellular iron concentration, approximately 50 mg of the center of each tissue sample was cut out from samples stored at − 80 °C. Each tissue was homogenized in ice-cold T-PER Tissue Protein Extraction Reagent (potentially containing 25 mM bicine and 150 mM NaCl [pH 7.6]) (Thermo Fisher Scientific, Waltham, MA, USA), and 1 M hydrochloric acid was added to the supernatant to obtain a concentration of 0.01 M. The supernatant obtained by centrifugation at 10,000 rpm was used for measurements. Intracellular iron concentration was corrected for the actual amount of protein. Varioskan LUX (Thermo Fisher Scientific, Waltham, MA, USA) was used for data measurement.

### Measurement of ATP levels

Intracellular ATP level was measured using ''Tissue'' ATP assay Kit (Toyo B-Net Co., Ltd., Tokyo, Japan) based on the luciferase luminescence method according to the kit's instructions. For measurements, liver and skeletal muscle were preferentially collected after blood sampling, rapidly frozen in liquid nitrogen, and stored at − 80 °C. Briefly, approximately 20 mg of each tissue was homogenized in homogenate buffer on ice and supernatant was used for this assay. Varioskan LUX (Thermo Fisher Scientific, Waltham, MA, USA) was used for data measurement. The ATP level was normalized by mtDNA content. The mtDNA content was measured as described previously^51^.

### Histological analysis and Transmission Electron Microscopy (TEM)

To perform hematoxylin and eosin (H&E) staining, collected liver tissues were fixed using 10% neutral buffered formalin immediately. Then tissue sections were prepared by embedding in paraffin. To perform Oil Red O staining, frozen sections were prepared using the collected liver tissue. These staining procedures were performed according to standard methods. Photographs were taken with a microscope camera (Moticam 1080; Shimadzu RIKA, Tokyo, Japan). ImageJ, version 1.52a was used to describe the scale bar. For electron microscope analysis, collected gastrocnemius tissues were fixed immediately using 2.5% glutaraldehyde in 0.1 M phosphate buffer (pH 7.4). To make tissue sections, these samples were dehydrated with ethanol, embedded in epoxy resin and then sliced using a microtome with a diamond knife. Uranyl acetate and lead citrate were prepared, and tissue sections collected on mesh grids were stained. Gastrocnemius morphology and mitochondria were observed using an electron microscope (1230 EXII; JEOL, Tokyo, Japan). Images of tissue sections were taken with a Gatan bio scan camera model 792.

### Cell culture

Hepatocytes were collected from five-weeks-old male C57BL/6J mice (Japan SLC, Inc., Shizuoka, Japan) given MF diet (Oriental Yeast Co., Ltd., Tokyo, Japan). Mice were anaesthetised, and the liver was perfused with a liver perfusion medium (Gibco) and then with a liver digest medium (Gibco) (both flow rate 5 mL/min). The liver was subdivided in L-15 medium (Gibco) to separate hepatocytes. Hepatocytes were washed twice with L-15 medium and then washed three times with hepatocyte wash medium (Gibco). Hepatocytes were isolated by centrifugation at 500 rpm for 3 min at 4 ℃, resuspended in William's Medium E (Gibco), supplemented with 10% FBS, 100 units/mL penicillin and 100 µg/mL streptomycin (Wako, Osaka, Japan), and plated at a density of 8 × 10^4^ cells/cm^2^. Plates coated with rat tail collagen I was used. Cells were incubated under conditions of 37 ℃ and 5% CO_2_. After 4 h, the medium was replaced with Dulbecco's modified eagle medium (DMEM) supplemented with 10% (v/v) FBS, 100 units/mL penicillin, 100 µg/mL streptomycin, 0.5 µM dexamethasone, and 0.4 µM insulin. After 18 h, the medium was replaced with assay medium and further incubated for 24 h. The replacement media were DMEM containing 10% (v/v) FBS, 100 units/mL penicillin, 100 µg/mL streptomycin and 0.05% dimethyl sulfoxide (DMSO), either supplemented with or without SFC. Cells were harvested, and total RNA was isolated using Sepasol-RNA I Super G (Nacalai tesque, Kyoto, Japan) according to the kit’s instructions, and used for evaluation of the expression of genes associated with mitochondrial ETC, heme and Fe–S cluster synthesis.

### Statistical analysis

Graph Pad Prism, version 8.4.3 was used for statistical analysis. Grubbs test was used to identify outliers. Outliers were excluded from the original data. One-way analysis of variance followed by post-hoc Holm–Sidak’s test was performed to determine statistical significance. This test was performed using HF or Vehicle as a control. Values are expressed as mean ± SEM. Results showing two-tailed *p*-values < 0.05 were considered statistically significant.

## Supplementary Information

Supplementary Information.

## Data Availability

Microarray data set have been deposited in NCBI's Gene Expression Omnibus and are accessible through GEO Series accession number GSE161644 (https://www.ncbi.nlm.nih.gov/geo/query/acc.cgi?acc=GSE161644) and GSE161646 (https://www.ncbi.nlm.nih.gov/geo/query/acc.cgi?acc=GSE161646). The datasets generated and analysed during the current study are available from the corresponding author on reasonable request.

## References

[1] Aigner E, Feldman A, Datz C (2014). Obesity as an emerging risk factor for iron deficiency. Nutrients.

[2] Forbes JM, Cooper ME (2013). Mechanisms of diabetic complications. Physiol. Rev..

[3] Wenzel BJ, Stults HB, Mayer J (1962). Hypoferraemia in obese adolescents. Lancet Lond. Engl..

[4] Siddique A, Nelson JE, Aouizerat B, Yeh MM, Kowdley KV (2014). Iron deficiency in patients with nonalcoholic fatty liver disease is associated with obesity, female sex, and low serum hepcidin. Clin. Gastroenterol. Hepatol. Off. Clin. Pract. J. Am. Gastroenterol. Assoc..

[5] Gong L (2014). Weight loss, inflammatory markers, and improvements of iron status in overweight and obese children. J. Pediatr..

[6] Tussing-Humphreys LM (2010). Decreased serum hepcidin and improved functional iron status 6 months after restrictive bariatric surgery. Obesity.

[7] Bartholmey SJ, Sherman AR (1985). Carnitine levels in iron-deficient rat pups. J. Nutr..

[8] Davis MR (2012). Enhanced expression of lipogenic genes may contribute to hyperglycemia and alterations in plasma lipids in response to dietary iron deficiency. Genes Nutr..

[9] Datz C, Felder TK, Niederseer D, Aigner E (2013). Iron homeostasis in the metabolic syndrome. Eur. J. Clin. Invest..

[10] Stugiewicz M (2016). The influence of iron deficiency on the functioning of skeletal muscles: Experimental evidence and clinical implications. Eur. J. Heart Fail..

[11] Hoes MF (2018). Iron deficiency impairs contractility of human cardiomyocytes through decreased mitochondrial function. Eur. J. Heart Fail..

[12] de Mello AH, Costa AB, Engel JDG, Rezin GT (2018). Mitochondrial dysfunction in obesity. Life Sci..

[13] Wei Y, Rector RS, Thyfault JP, Ibdah JA (2008). Nonalcoholic fatty liver disease and mitochondrial dysfunction. World J. Gastroenterol..

[14] Camaschella C (2015). Iron-deficiency anemia. N. Engl. J. Med..

[15] Torrance JD, Charlton RW, Schmaman A, Lynch SR, Bothwell TH (1968). Storage iron in `muscle’. J. Clin. Pathol..

[16] Padda RS (2014). A high-fat diet modulates iron metabolism but does not promote liver fibrosis in hemochromatotic Hjv−/− mice. Am. J. Physiol. Gastrointest. Liver Physiol..

[17] Satoh A (2013). Sirt1 extends life span and delays aging in mice through the regulation of Nk2 homeobox 1 in the DMH and LH. Cell Metab..

[18] García-Ruiz I (2014). High-fat diet decreases activity of the oxidative phosphorylation complexes and causes nonalcoholic steatohepatitis in mice. Dis. Model. Mech..

[19] Li X, Higashida K, Kawamura T, Higuchi M (2018). Time course of decrease in skeletal muscle mitochondrial biogenesis after discontinuation of high-fat diet. J. Nutr. Sci. Vitaminol. (Tokyo).

[20] Ahmed U, Latham PS, Oates PS (2012). Interactions between hepatic iron and lipid metabolism with possible relevance to steatohepatitis. World J. Gastroenterol. WJG.

[21] Orr JS (2014). Obesity alters adipose tissue macrophage iron content and tissue iron distribution. Diabetes.

[22] Jiang S (2019). Long-term high-fat diet decreases hepatic iron storage associated with suppressing TFR2 and ZIP14 expression in rats. FASEB J..

[23] Sherman AR, Guthrie HA, Wolinsky I, Zulak IM (1978). Iron deficiency hyperlipidemia in 18-day-old rat pups: Effects of milk lipids, lipoprotein lipase, and triglyceride synthesis. J. Nutr..

[24] Sherman AR, Bartholmey SJ, Perkins EG (1982). Fatty acid patterns in iron-deficient maternal and neonatal rats. Lipids.

[25] Sherman AR (1978). Lipogenesis in iron-deficient adult rats. Lipids.

[26] Muckenthaler MU, Rivella S, Hentze MW, Galy B (2017). A red carpet for iron metabolism. Cell.

[27] Barupala DP, Dzul SP, Riggs-Gelasco PJ, Stemmler TL (2016). Synthesis, delivery and regulation of eukaryotic heme and Fe–S cluster cofactors. Arch. Biochem. Biophys..

[28] Alam MM, Lal S, FitzGerald KE, Zhang L (2016). A holistic view of cancer bioenergetics: mitochondrial function and respiration play fundamental roles in the development and progression of diverse tumors. Clin. Transl. Med..

[29] Bitar M, Weiner M (1983). Modification of age-induced changes in heme and hemoproteins by testosterone in male rats. Mech. Ageing Dev..

[30] Hayashi J (1994). Nuclear but not mitochondrial genome involvement in human age-related mitochondrial dysfunction. Functional integrity of mitochondrial DNA from aged subjects. J. Biol. Chem..

[31] Saitoh S (2018). 5-aminolevulinic acid (ALA) deficiency causes impaired glucose tolerance and insulin resistance coincident with an attenuation of mitochondrial function in aged mice. PLoS ONE.

[32] Alfadhel M (2011). Infantile cardioencephalopathy due to a COX15 gene defect: Report and review. Am. J. Med. Genet. A..

[33] Al-Hassnan ZN (2015). ISCA2 mutation causes infantile neurodegenerative mitochondrial disorder. J. Med. Genet..

[34] Xu W, Barrientos T, Andrews NC (2013). Iron and copper in mitochondrial diseases. Cell Metab..

[35] Atamna H, Killilea DW, Killilea AN, Ames BN (2002). Heme deficiency may be a factor in the mitochondrial and neuronal decay of aging. Proc. Natl. Acad. Sci. USA.

[36] Diaz F, Enríquez JA, Moraes CT (2012). Cells lacking rieske iron–sulfur protein have a reactive oxygen species-associated decrease in respiratory complexes I and IV. Mol. Cell. Biol..

[37] Cameron JM (2011). Mutations in iron–sulfur cluster scaffold genes NFU1 and BOLA3 cause a fatal deficiency of multiple respiratory chain and 2-oxoacid dehydrogenase enzymes. Am. J. Hum. Genet..

[38] Tajima K (2019). Mitochondrial lipoylation integrates age-associated decline in brown fat thermogenesis. Nat. Metab..

[39] McKie AT (2008). The role of Dcytb in iron metabolism: An update. Biochem. Soc. Trans..

[40] Sonnweber T (2012). High-fat diet causes iron deficiency via hepcidin-independent reduction of duodenal iron absorption. J. Nutr. Biochem..

[41] Shawki A (2015). Intestinal DMT1 is critical for iron absorption in the mouse but is not required for the absorption of copper or manganese. Am. J. Physiol. Gastrointest. Liver Physiol..

[42] Bacon BR, Farahvash MJ, Janney CG, Neuschwander-Tetri BA (1994). Nonalcoholic steatohepatitis: An expanded clinical entity. Gastroenterology.

[43] George DK (1998). Increased hepatic iron concentration in nonalcoholic steatohepatitis is associated with increased fibrosis. Gastroenterology.

[44] Fargion S (2001). Hyperferritinemia, iron overload, and multiple metabolic alterations identify patients at risk for nonalcoholic steatohepatitis. Am. J. Gastroenterol..

[45] Harrison SA, Bacon BR (2003). Hereditary hemochromatosis: Update for 2003. J. Hepatol..

[46] Thomas C, Thomas L (2002). Biochemical markers and hematologic indices in the diagnosis of functional iron deficiency. Clin. Chem..

[47] Skikne BS (2011). Improved differential diagnosis of anemia of chronic disease and iron deficiency anemia: A prospective multicenter evaluation of soluble transferrin receptor and the sTfR/log ferritin index. Am. J. Hematol..

[48] Dziegala M (2018). Iron deficiency as energetic insult to skeletal muscle in chronic diseases. J. Cachexia Sarcopenia Muscle.

[49] Watanabe M (2004). Bile acids lower triglyceride levels via a pathway involving FXR, SHP, and SREBP-1c. J. Clin. Invest..

[50] Folch J, Lees M, Stanley GHS (1957). A simple method for the isolation and purification of total lipides from animal tissues. J. Biol. Chem..

[51] Quiros PM, Goyal A, Jha P, Auwerx J (2017). Analysis of mtDNA/nDNA ratio in mice. Curr. Protoc. Mouse Biol..

