# Supplementary material for: Iron supplementation regulates the progression of high fat diet induced obesity and hepatic steatosis via mitochondrial signaling pathways
Source: Sci Rep. 2021 May 24;11:10753. doi: 10.1038/s41598-021-89673-8 (PMC8144192; doi:10.1038/s41598-021-89673-8)
Supplement: Supplementary file 1 — Supplementary Information. [file 41598_2021_89673_MOESM1_ESM.pdf]

Supplementary information

Iron supplementation regulates the progression of high fat diet induced obesity and hepatic steatosis via mitochondrial signaling pathways

Naho Kitamura [1,2], Yoko Yokoyama [1,2], Hiroki Taoka [1,2], Utana Nagano [1,2], Shotaro Hosoda [1,2], Tanon Taworntawat [1,2], Anna Nakamura [1,2], Yoko Ogawa [3], Kazuo Tsubota [2,3,*], and Mitsuhiro Watanabe [1,2,3,4,5,*]

[1] Systems Biology Program, Graduate School of Media and Governance, Keio University, 5322, Endo, Fujisawa, Kanagawa 252-0882, Japan

[2] Health Science Laboratory, Keio Research Institute at SFC, 5322, Endo, Fujisawa, Kanagawa 252-0882, Japan

[3] Department of Ophthalmology, Keio University School of Medicine, 35 Shinanomachi, Shinjuku-ku, Tokyo 160-8582, Japan

[4] Department of Environment and Information Studies, Keio University, 5322, Endo, Fujisawa, Kanagawa 252-0882, Japan

[5] Department of Internal Medicine, Keio University School of Medicine, 35 Shinanomachi, Shinjuku-ku, Tokyo 160-8582, Japan

*Corresponding Authors:

Kazuo Tsubota:

Email: tsubota@z3.keio.jp

Phone: +81-3-6384-2866

Mitsuhiro Watanabe:

Email: mitsuhiro.keio.hsl@gmail.com

Phone: +81-466-48-5099

ORCID

Naho Kitamura: https://orcid.org/0000-0001-6894-3046

Yoko Yokoyama: https://orcid.org/0000-0002-4249-004X

Kazuo Tsubota: https://orcid.org/0000-0002-8874-7111

Mitsuhiro Watanabe: https://orcid.org/0000-0002-4500-780X

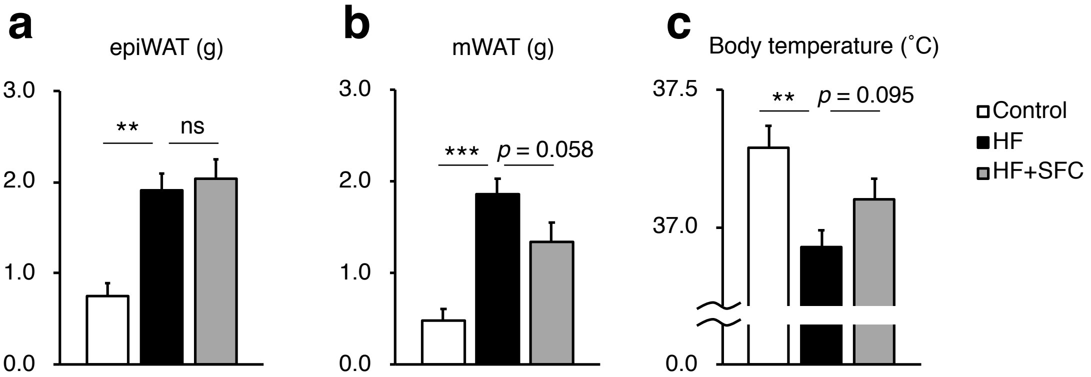

Supplementary Figure S1.

a-c, Male C57BL/6J mice of 6 weeks of age were fed with an open source control diet (Control; 10% kcal fat), a high-fat diet (HF; 60% kcal fat), or a high-fat diet supplemented with sodium ferrous citrate (HF+SFC) for 15 weeks.

(**a-b**) (**a**) Weight of epididymal WAT (epiWAT); and (**b**) mesenteric WAT (mWAT) (n = 5-6).

(**c**) Rectal body temperature in mice fed 7 weeks with the different diets. (n = 11-12).

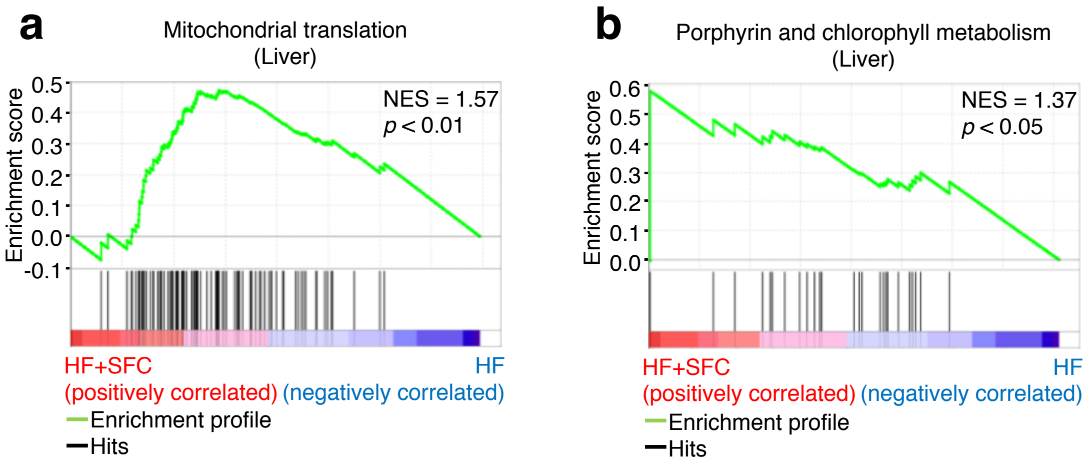

Supplementary Figure S2.

(**a, b**) Male C57BL/6J mice of 6 weeks of age were fed with each diet for 15 weeks as specified in Supplementary Figure S1. For each group, a mix of all sample cDNAs was used. Enrichment plots of genes upregulated in HF+SFC liver (compared to HF). (**a**) Reactome database; or (**b**) Kyoto Encyclopedia of Genes and Genomes (KEGG) database was used. Analysis was performed by Gene Set Enrichment Analysis (GSEA). NES: normalized enrichment score.

Supplementary Table S1. The list of 51 upregulated mitochondrial genes in the liver and skeletal muscle of HF+SFC revealed in Figure 6a.

Gene Symbol	Description	Entrez Gene ID
1110001J03Rik	RIKEN cDNA 1110001J03 gene	66117
Abcb9	ATP-binding cassette, sub-family B (MDR/TAP), member 9	56325
Abcd3	ATP-binding cassette, sub-family D (ALD), member 3	19299
Acly	ATP citrate lyase	104112
Acsm2	acyl-CoA synthetase medium-chain family member 2	233799
Ak4	adenylate kinase 4	11639
Akr1b10	aldo-keto reductase family 1, member B10 (aldose reductase)	67861
Aldh7a1	aldehyde dehydrogenase family 7, member A1	110695
Apool	apolipoprotein O-like	68117
Atp5sl	ATP5S-like	66349
Bola1	bolA-like 1 (E. coli)	69168
Ccdc127	coiled-coil domain containing 127	67433
Cdc25c	cell division cycle 25C	12532
Cep89	centrosomal protein 89	72140
Coq10a	coenzyme Q10 homolog A (yeast)	210582
Csl	citrate synthase like	71832
Cyp24a1	cytochrome P450, family 24, subfamily a, polypeptide 1	13081
Dnajc11	DnaJ (Hsp40) homolog, subfamily C, member 11	230935
Gbas	glioblastoma amplified sequence	14467
Grsf1	G-rich RNA sequence binding factor 1	231413
Guf1	GUF1 GTPase homolog (S. cerevisiae)	231279
Lipt1	lipoyltransferase 1	623661
Malsu1	mitochondrial assembly of ribosomal large subunit 1	75593
Marchf5	membrane-associated ring finger (C3HC4) 5	69104
Mdh1	malate dehydrogenase 1, NAD (soluble)	17449

Mrpl3	mitochondrial ribosomal protein L3	94062
Mthfd2l	methylenetetrahydrofolate dehydrogenase (NADP+ dependent) 2-like	665563
Mto1	mitochondrial translation optimization 1 homolog (S. cerevisiae)	68291
Mutyh	mutY homolog (E. coli)	70603
Ndor1	NADPH dependent diflavin oxidoreductase 1	78797
Ndufb2	NADH dehydrogenase (ubiquinone) 1 beta subcomplex, 2	68198
Ndufb9	NADH dehydrogenase (ubiquinone) 1 beta subcomplex, 9	66218
Oxsm	3-oxoacyl-ACP synthase, mitochondrial	71147
Pak7	p21 protein (Cdc42/Rac)-activated kinase 7	241656
Pick1	protein interacting with C kinase 1	18693
Pif1	PIF1 5'-to-3' DNA helicase homolog (S. cerevisiae)	208084
Ppox	protoporphyrinogen oxidase	19044
Rmdn1	regulator of microtubule dynamics 1	66302
Rps18	ribosomal protein S18	20084
Rsad1	radical S-adenosyl methionine domain containing 1	237926
Sdha	succinate dehydrogenase complex, subunit A, flavoprotein (Fp)	66945
Sirt5	sirtuin 5	68346
Slc25a14	solute carrier family 25 (mitochondrial carrier, brain), member 14	20523
Slc25a31	solute carrier family 25 (mitochondrial carrier; adenine nucleotide translocator), member 31	73333
Slc25a48	solute carrier family 25, member 48	328258
Slc25a54	solute carrier family 25, member 54	74686
Slmo1	slowmo homolog 1 (Drosophila)	225655
Star	steroidogenic acute regulatory protein	20845
Sucla2	succinate-Coenzyme A ligase, ADP-forming, beta subunit	20916
Tufm	Tu translation elongation factor, mitochondrial	233870
Uqcrq	ubiquinol-cytochrome c reductase, complex III subunit VII	22272

Supplementary Table S2. The composition of diets the control, high-fat (HF), and iron supplemented HF (HF+SFC) diets used in this study.

	**Control (D12450B)**		**HF (D12492)**		**HF (D12492) + SFC**	
	gm%	kcal%	gm%	kcal%	gm%	kcal%
Protein	19.2	20	26	20	26	20
Carbohydrate	67.3	70	26	20	26	20
Fat	4.3	10	35	60	35	60
Total		100		100		100
kcal/gm	3.85		5.24		5.24	
Ingredient	gm	kcal	gm	kcal	gm	kcal
Casein	200	800	200	800	200	800
L-Cystine	3	12	3	12	3	12
Corn Starch	315	1260	0	0	0	0
Maltodextrin	35	140	125	500	125	500
Sucrose	350	1400	68.8	275	68.8	275
Cellulose	50	0	50	0	50	0
Soybean Oil	25	225	25	225	25	225
Lard	20	180	245	2205	245	2205
Mineral Mix S10026	10	0	10	0	10	0
DiCalcium Phosphate	13	0	13	0	13	0
Calcium Carbonate	5.5	0	5.5	0	5.5	0
Potassium Citrate, 1 H2O	16.5	0	16.5	0	16.5	0
Vitamin Mix V10001	10	40	10	40	10	40
Choline Bitartrate	2	0	2	0	2	0
FD&C Yellow Dye #5	0.05	0	0	0	0	0
FD&C Blue Dye #1	0	0	0.05	0	0.05	0
Sodium Ferrous Citrate	0	0	0	0	0.18	0
Total	1055.05	4057	773.85	4057	774.03	4057

Supplementary Table S3. Composition of micronutrients present in 4057 kcal of diets.

	Control (D12450B)	HF (D12492)	HF (D12492) +SFC
**Minerals (mg)**			
Fe	37	37	56
Mo	1.6	1.6	1.6
Cr	2.0	2.0	2.0
Cu	6.0	6.0	6.0
Mn	59	59	59
I	0.2	0.2	0.2
Fl	0.9	0.9	0.9
Se	0.16	0.16	0.16
Zn	29	29	29
**Minerals (g)**			
Na	1.0	1.0	1.0
Cl	1.6	1.6	1.6
Mg	0.5	0.5	0.5
S	0.33	0.33	0.33
**Vitamins**			
Vit A (IU)	4000	4000	4000
Vit D3 (IU)	1000	1000	1000
Vit E (IU)	50	50	50
Menadione (mg)	0.5	0.5	0.5
Biotin (mg)	0.2	0.2	0.2
Vit B12 (μg)	10	10	10
Folic Acid (mg)	2	2	2
Niacin (mg)	30	30	30
Pantothenic Acid (mg)	16	16	16
Vit B6 (mg)	7	7	7
Vit B2 (mg)	6	6	6
Vit B1 (mg)	6	6	6

Supplementary Table S4. Primer sequences for qRT-PCR analysis.

Gene	Forward Primer (5'→3')	Reverse Primer (5'→3')
Abcb7	TGCAGTTACACGGTGGAGAA	CAAAATTCAGCATTGCCAGA
Acox1	TTCTACCAATCTGGCTGCAC	GTGGGTGGTATGGTGTCGTA
Acsl3	GCGAGAAGGATTCCAAGACTGG	GAAGAGTAGCCGATTCGGCATC
Actb	CATCCGTAAAGACCTCTATGCCAAC	ATGGAGCCACCGATCCACA
Atp5o	AGGCCCTTTGCCAAGCTT	TTCTCCTTAGATGCAGCAGAGTACA
B2m	TGCTACTCGGCGCTTCAGTC	AGGCGGGTGGAACTGTGTTAC
Bola3	CCTCGAGCCACAGCTATTCA	CGTGCATGCCCTTGATCTCT
Coq10a	CAAGCGAAAGGCTTACTCGGAG	CCAGTGACTTCTTACACCACGG
Cox10	TTCCTGCTTACATCCCTTGG	TTCATGTTTGAGTCGAACGG
Cox6b1	ATGTCTCCGTGTGTGAGTGG	GATCTTCCCAGGAAATGTGC
Cpt1a	TGGGCTACTCAGAGGATGG	AAGGTGTCAAATGGGAAGG
Cpt1b	GGATGTTCGAGATGCACAGC	GGAAGCTGTAGAGCATGGGCCG
Cyc1	GCTTCCAGGTGCAAGTGCT	CAGACTTCGAGGACAAGGACA
Cycs	GCAAGCATAAGACTGGACCAAA	TTGTTGGCATCTGTGTAAGAGAATC
Echs1	AGCCTGTAGCTCACTGTTGTC	ATGTACTGAAAGTTAGCACCCG
Fastkd2	ATGAATAGCAAAGCACGTTCCT	TAGCCGATGGTTTCTTGGGTT
Hadh	TTCCAGAGGCTGGACAAGTTCG	GCCAGCAAATCGGTCTTGTCTG
Hamp1	CCTATCTCCATCAACAGAT	TGCAACAGATACCACACTG
Hmbs	ACTCTGCTTCGCTGCATTG	AGTTGCCCATCTTTCATCACTG
Isca2	GGCCTTGTCCCTAACTGCC	TGACCCTTCGGTGATTTCCAG
Lcad	ACGATCTGTCTTGCGATCAG	ACATGTGGGAGTACCCGATT
Mcad	AGGGTTTAGTTTTGAGTTGACGG	CCCCGCTTTTGTCATATTCCG
Mlycd	CTGAGGATCTGCTCGGAAGCTTTG	TGGATGGCTGACAGCAGCCTCAA
mt-Co1	ACACAACTTTCTTTGATCCCG	AGAATCAGAACAGATGCTGG
mt-Cyb	CCATTCTACGCTCAATCCCCA	AGGCTTCGTTGCTTTGAGGTA
Ndufa1	GTCCACTGCGTACATCCACA	ATCGCGTTCCATCAGATACC
Nrf1	TCGGAGCACTTACTGGAGTC	CTGGTACATGCTCACAGGGA
Pgc1a	AAGGGCCAAACAGAGAGAGA	GCGTTGTGTCAGGTCTGATT
Pgc1b	AGATGAAGATCCAAGCTGCCCACA	TCCTCCTCCATTGGCTTGTATGGA

Sdhc	AGTTTGTGCTTGTCTTCCCG	CACTCCAGACAGCCAGACCT
Sdhd	CATGGCGGTTCTCTTAAAGC	TGACACATAAGCGGGTCTGA
Tfam	ATGTCTCCGGATCGTTTCAC	CCAAAAAGACCTCGTTCAGC
Ucp3	CTCTGCACTGTATGCTGAAGATG	CACGTTCCAAGCTCCCAGA
Uqcrfs1	ATGTGAAGCGACCCTTCCT	GGAAAAACGGACAGAAGCAG
Vlcad	GGCCAAGCTGGTGAAACACAAGAA	ACAGAACCACCACCATGGCATAGA
